# Correction: Shedding a new light on Huntington’s disease: how blood can both propagate and ameliorate disease pathology

**DOI:** 10.1038/s41380-020-0850-1

**Published:** 2020-07-24

**Authors:** Marie Rieux, Melanie Alpaugh, Giacomo Sciacca, Martine Saint-Pierre, Maria Masnata, Hélèna L. Denis, Sébastien A. Lévesque, Frank Herrmann, Chantal Bazenet, Alexandre P. Garneau, Paul Isenring, Ray Truant, Abid Oueslati, Peter V. Gould, Anne Ast, Erich E. Wanker, Steve Lacroix, Francesca Cicchetti

**Affiliations:** 1 Centre de Recherche du CHU de Québec—Université Laval, Axe Neurosciences, 2705 boulevard Laurier, Québec, QC G1V 4G2 Canada; 2 Département de Médecine Moléculaire, Université Laval, 1050 avenue de la Médecine, Québec, QC G1V 0A6 Canada; 3 Département de Psychiatrie & Neurosciences, Université Laval, 1050 avenue de la Médecine, Québec, QC G1V 0A6 Canada; 4 Evotec SE, Essener Bogen 7, 22419 Hamburg, Germany; 5 L’Hôtel-Dieu de Québec—Université Laval, Axe Endocrinologie et Néphrologie, 10 rue McMahon, Québec, QC G1R 2J6 Canada; 6 Ecole de Kinésiologie et des Sciences de l’Activité Physique, Université de Montréal, 2100 boulevard Édouard-Montpetit, Montréal, QC H3T 1J4 Canada; 7 Department of Biochemistry and Biomedical Research, McMaster University, 1280 Main Street West, Hamilton, ON L8S 4L8 Canada; 8 Département d’Anatomopathologie et de Cytologie, Centre Hospitalier Affilié Universitaire de Québec, Hôpital de l’Enfant-Jésus, 1401 18ème rue, Québec, QC G1J 1Z4 Canada; 9 Neuroproteomics, Max Delbrueck Center for Molecular Medicine, 13125 Berlin, Germany; 10 Berlin Institute of Health, 10178 Berlin, Germany

**Keywords:** Neuroscience, Diseases

Correction to: *Molecular Psychiatry*

10.1038/s41380-020-0787-4 published online 08 June 2020

This article was originally published under Nature Research’s License to Publish, but has now been made available under a [CC BY 4.0] license. The PDF and HTML versions of the article have been modified accordingly.

